# Protocol of a parallel, randomized controlled trial on the effects of a novel personalized nutrition approach by artificial intelligence in real world scenario

**DOI:** 10.1186/s12889-023-16434-9

**Published:** 2023-09-02

**Authors:** Jingyuan Feng, Hongwei Liu, Shupeng Mai, Jin Su, Jing Sun, Jianjie Zhou, Yingyao Zhang, Yinyi Wang, Fan Wu, Guangyong Zheng, Zhenni Zhu

**Affiliations:** 1 School of Public Health, Fudan University, Shanghai, China; 2 Division of Health Risk Factors Monitoring and Control, Shanghai Municipal Center for Disease Control and Prevention, Shanghai, China; 3 Chinese Center for Disease Control and Prevention, National Institute for Nutrition and Health, Beijing, China; 4 Basebit (Shanghai) Information Technology Co., Ltd, Shanghai, China; 5 Department of Nutrition and Food Science, Education, and Human Development, Steinhardt School of Culture, New York University, New York, USA; 6 Institute of Interdisciplinary Integrative Medicine Research, Shanghai University of Traditional Chinese Medicine, Shanghai, China

**Keywords:** Personalized nutrition, Nutrition intervention, Artificial intelligence, Smartphone applet, Real world

## Abstract

**Background:**

Nutrition service needs are huge in China. Previous studies indicated that personalized nutrition (PN) interventions were effective. The aim of the present study is to identify the effectiveness and feasibility of a novel PN approach supported by artificial intelligence (AI).

**Methods:**

This study is a two-arm parallel, randomized, controlled trial in real world scenario. The participants will be enrolled among who consume lunch at a staff canteen. In Phase I, a total of 170 eligible participants will be assigned to either intervention or control group on 1:1 ratio. The intervention group will be instructed to use the smartphone applet to record their lunches and reach the real-time AI-based information of dish nutrition evaluation and PN evaluation after meal consumption for 3 months. The control group will receive no nutrition information but be asked to record their lunches though the applet. Dietary pattern, body weight or blood pressure optimizing is expected after the intervention. In phase II, the applet will be free to all the diners (about 800) at the study canteen for another one year. Who use the applet at least 2 days per week will be regarded as the intervention group while the others will be the control group. Body metabolism normalization is expected after this period. Generalized linear mixed models will be used to identify the dietary, anthropometric and metabolic changes.

**Discussion:**

This novel approach will provide real-time AI-based dish nutrition evaluation and PN evaluation after meal consumption in order to assist users with nutrition information to make wise food choice. This study is designed under a real-life scenario which facilitates translating the trial intervention into real-world practice.

**Trial registration:**

This trial has been registered with the Chinese Clinical Trial Registry (ChiCTR2100051771; date registered: 03/10/2021).

## Background

Suboptimal dietary pattern is a major contributing factor to the growing incidence of non-communicable diseases (NCDs) in China, which is characterized by a high intake of fat and sodium and a low intake of vegetables and fruits [1, 2]. The rapid modernization of the restaurant and packaged food industry has aggravated unhealthy dietary pattern [3]. China has set a nationwide target to reduce the intake of dietary oil, salt, and sugar, as well as promoting nutrition education to help the public adopt healthier eating habits [4]. However, recent data revealed that in comparison to the number of registered dietitians (RDs) and registered dietetic technicians (DTRs) in developed countries, China had a much lower ratio, with only 67 RDs/DTRs per 10 million people, which is far from meeting the massive needs of nutrition guidance [5].

PN (personalized nutrition) is a field that utilizes human individuality to develop nutrition approaches that prevent, manage, and treat diseases and optimize health [6]. Previous studies have suggested that smartphones with built-in AI (artificial intelligence)-based algorithms are cost-effective, flexible, visually appealing and engaging, which could approach PN guidance and therapeutics provided by real healthcare professionals [7, 8].

Substantial evidence indicates that randomized controlled trials (RCTs) hardly shift into real-world practice [9]. In most long-term weight loss studies in humans, favorable results were achieved during the intervention but hard to keep after returning to normal life [10]. In contrast, real world studies allow participants to follow their daily routines and enjoy real-life meals on wills, which might facilitate a trial becoming a real-world practice [11].

We have developed a PN approach with an AI-based smartphone applet, which aims to help provide the information of dish nutrition evaluation and PN evaluation after meal consumption for the mass of people under the circumstance of dietitian shortage in China. This study is to identify the effectiveness and feasibility of this novel approach in a parallel, randomized, controlled trial in real world scenario.

## Methods

### Setting and study design

This trial is a two-arm parallel, randomized, controlled study in real-world situations. This smartphone applet with built-in AI-based algorithm only takes effect where dishes prepared by recipes with quantity (in China, same dish prepared at different home are different in cooking but dishes in central kitchen or food factory are prepared strictly following standard cooking procedure [12]). Participants are enrolled sequentially until the sample size reaches requirement. This study will be carried out in a pilot company employing 3000 people and food is prepared by the central kitchen at staff canteen. All participants will choose and consume meals on their own wills during the study. Weight and blood pressure measuring tools placed at the canteen can be available to participants; therefore, they can conveniently measure and record anthropometric indicators through the applet.

#### Phase I

The employees will be recruited to dine in the canteen for 3 months, assigned to the intervention or control group randomly. Both groups will be asked to use the applet to record their lunch each weekday during the study. After 1 week of run-in period, the intervention group will be able to access the information of dish nutrition evaluation and PN evaluation after meal consumption, while the control group will not. Both groups will be followed up by researchers on the same time schedules for the outcome measurements.

#### Phase II

Full-functioned applet will be available for use by all the diners (about 800) for another 1 year. Who use the applet at least 2 days per week will be regarded as the intervention group while the others will be the control group. During this phase, metabolic indicators from the annual physical examination will be provided by the company.

**Fig. 1:**
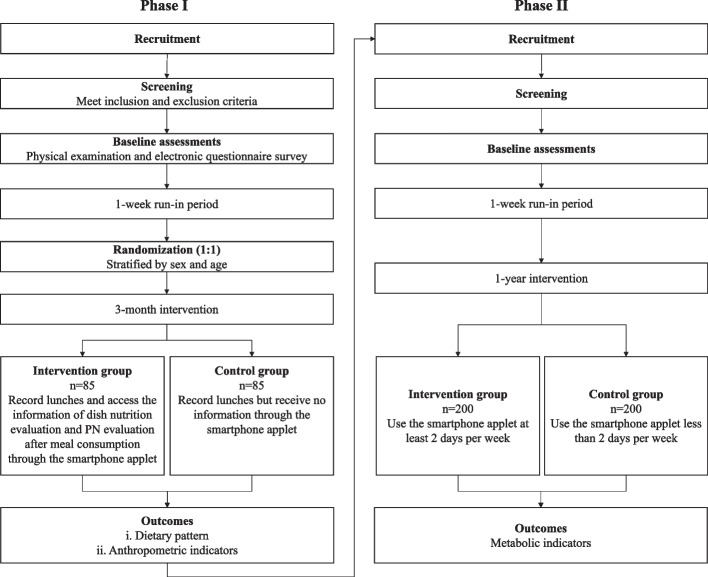
Study design of the trial

Figure 1 outlines the study design and Table 1 presents the visits and data collection schedule.

**Table 1:** Visits and data collection schedule

	Phase I (3months)			Phase II (1year)		
	Screening	Run-in	Follow-up	Screening	Run-in	Follow-up
Informed consent	√			√		
Questionnaire		√	√		√	√
Dietary record		√	√		√	√
Anthropometric indicators		√	√		√	√
Metabolic indicators					√	√

### Sample size

In Phase I, we conservatively assumed that the percentage of energy intake from fat as a key indicator will reduce by 6% after intervention. According to the findings from China National Nutrition Survey (CNNS), the SD of that indicator was 11% [13]. Thus, 85 participants in each group are expected to be enrolled with a 5% significance level, a 90% statistical power and a 15% dropout rate. In Phase II, assuming that the prevalence of metabolic syndrome after intervention is approximately 20% based on the available research evidence [14], we would need 200 participants in each group to have sufficient power to detect the given effect size.

### Procedure

#### Inclusion and exclusion criteria

Men and women will be eligible to participate if they (1) are more than 18 years old, (2) are healthy in appearance, (3) promise to have lunch at the staff canteen during the study period, (4) agree to record food consumption of each meal on the applet. Exclusion criteria include: (1) planning to change physical activity habits during the course of study; (2) unable to follow a regular diet (e.g. on diet).

#### Recruitment and follow-up

##### Pilot

To establish the feasibility and acceptability of current protocol, we recruited 94 employees to perform a 3-week, single-arm preliminary pilot trial and users tested this applet (data collection from February 2022 to March 2022).

##### Phase I

Recruitment and physical examination are planned to conduct at regular intervals during 3 months. Researchers ask employees about participation in the study, collect written informed consent from participants, instruct them to use the applet as well as measure their anthropometric indicators in a standardized way. Thereafter, participants complete demographic questions (birthdate, sex and occupation) through the applet, as well as answer questions regarding their family history of NCDs, physical fitness, drug use, physical activity level (PAL), sleep, smoking status, alcohol consumption, dietary habits and nutrition service needs. The follow-up questionnaire will be repeated at the end of the phase I and questions about user experience of this applet will be added to it.

##### Phase II

The intervention will last for another 1 year. All the diners will access the full-functioned applet. Metabolic data will be collected from the annual physical examination by the company. Participants will complete the same electronic questionnaire at pre- and post-intervention.

#### Randomization and blinding

Following baseline measurements, the newly-enrolled participants will be randomized at a ratio of 1:1 to an intervention or control group. Randomization is stratified by sex and age. The trial is conducted as a double-blind study. The researchers are not blinded to allocation due to the nature of the intervention strategy, however the field investigators and participants will be blinded throughout the study, ensuring allocation concealment.

### The intervention

**Fig. 2:**
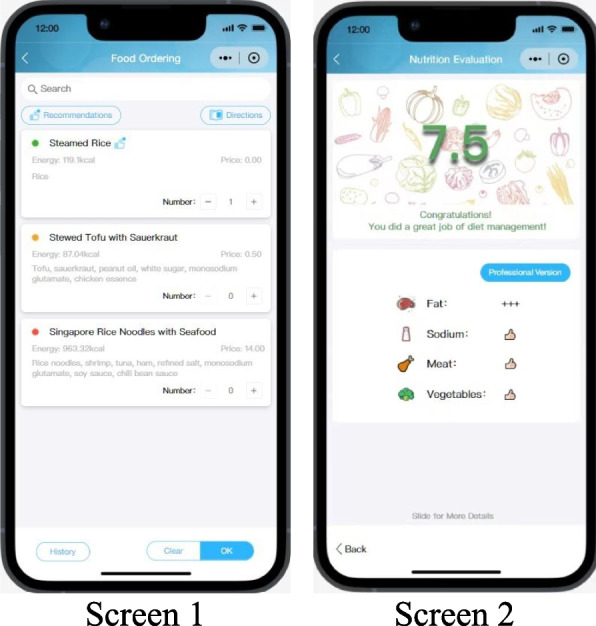
User interfaces of the AI-based PN management applet. Screen 1 demonstrates dish nutrition evaluation. Screen 2 shows PN evaluation after meal consumption

The intervention is to provide AI-based dish nutrition evaluation and PN evaluation after meal consumption at the study canteen through the applet (Fig. 2). The applet based on privacy-preserving computing platform has two interfaces: a phone-based client and a web-based data management system. For users, this applet is a WeChat mini-program which has ease of use, high acceptance and little memory [15]. Similar to Facebook, WeChat is a very popular social software in China. The mini-program relying on WeChat can achieve health functions and provide native app-like experiences without leaving WeChat interface. For researchers, the web-based system is used to facilitate central management including user registration, recipe preparation, menu administration and information storage. The built-in AI algorithm is applied in data operation and processing to support iterative calculation.

#### Dish nutrition evaluation

Dish nutrition evaluation is used to determine whether the content of fat, sodium and sugar in dish is benefit for health.

Two independent datasets are used for our study. First is the Chinese food composition database [16], which is publicly available. This database includes nutritional values for over 1110 food items and corresponding food groups. Second, we create a recipe dataset of all the dishes supplied in this canteen. The recipe includes the raw weights of materials (including ingredients and condiments) and their edible proportion, as well as gross and single-portion cooked weights of each dish. Weight measurements are conducted by field investigators with expertise in nutrition under the same standard criteria.

We construct a full dish/nutrition dataset by connecting the recipe dataset to Chinese food composition database. Thus, the food groups, energy and nutrients for each dish are automatically calculated using Algorithm 1. Food groups are classified as cereals & tubers (grains, potatoes and tubers), vegetables (excluding legumes), fruits (including citrus), livestock and poultry meat, eggs and products, seafood, dairy, nuts, soybeans and products, cooking oil, salt and sugar. Plant food stands for cereals & tubers, vegetables, fruits, soybeans and products. Animal food stands for livestock and poultry meat, seafood, eggs and products. Nutrients include protein, fat, carbohydrate, cholesterol, sodium, calcium, iron, zinc and vitamin C.

**Figure:**
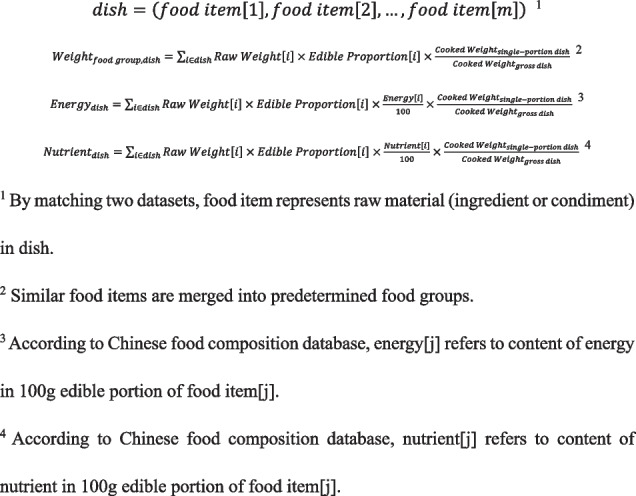
**Algorithm 1** Calculation of food groups, energy and nutrients for each dish

The intervenors can browse and choose dishes on the ordering interface of the applet. Colored dots are displayed next to the dishes’ names by a “traffic light” approach to indicate whether the dishes are benefit for health. The judgement of three colors is based on the contents of fat, sodium and sugar (green = reaching the dietary recommendations, yellow = between the recommendations and average intakes among Chinese population [13], red = above the upper limit of intakes). The dietary recommendations in this study are: no more than 8 g fat, 500 mg sodium and 4.5 g sugar in 100 g dish (raw weight except for condiments). The cutoffs of nutrient contents according to the definition of “traffic lights” are listed in Table 2.

**Table 2:** The cutoffs of nutrient contents for dish nutrition evaluation^a^

Nutrient content	I	II	III
Fat (g/100g^b^)	< 8	8–20	> 20
Sodium (mg/100g^b^)	< 500	500–1000	> 1000
Sugar (g/100g^b^)	< 4.5	4.5–9	> 9

^a^Green light for the dish represents all three indices within the range in the I column, red indicates at least 1 index within the range in the III column and yellow includes all the others

^b^100 g refers to 100 g edible portion of dish

#### PN evaluation after meal consumption

PN evaluation after meal consumption is used to illustrate whether food intakes are inadequate, adequate or excessive.

A meal may consist of different dishes in varying portions. To obtain the actual intake of each chosen dish, nutritional values are multiplied by portions and non-discarded proportion that are input by participants on the ordering interface. Subsequently, to calculate the meal consumption, the consumptions of corresponding food groups, energy and nutrients from different dishes are summed up (Algorithm 2). The whole-day consumption can be further calculated according to self-reported contribution of three meals to total daily food intake.

**Figure:**
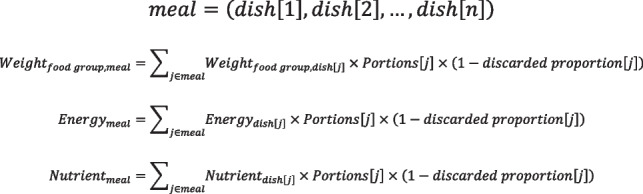
**Algorithm 2** Calculation of food groups, energy and nutrients for meal

According to individual’s biological profile and total energy expenditure (TEE), the specific recommended intake of the individual will be determined. TEE can be calculated using resting energy expenditure (REE) multiplied by PAL [17]. REE is estimated by Schofield equation regarding age, sex and body weight [18]. PAL is categorized into 1.5 for light, 1.75 for moderate, and 2.0 for vigorous physical activity [19]. Based on Chinese Food Recommendation [20], TEE ranging from 1000 kcal/day to 3000 kcal/day can be divided to 11 ranks and nutrition needs for a balanced diet pattern vary with different TEE ranks. Taking self-reported contribution of three meals to total daily food intake into consideration, recommendations of food groups and nutrients per meal can be assessed according to Chinese Dietary Guidelines [21] and Chinese Dietary Reference Intakes (DRIs) [19], respectively.

By comparison with recommended intake, the PN evaluation regarding actual intake will be provided for participants. The key evaluation indices for meal consumption are described in Table 3, including fat, sodium, meat and vegetables. For each index, we assign a health score with a corresponding mark to illustrate the comparisons between the current consumption and the national dietary recommendations. For example, a mark of three plus (+ + +) for fat indicates that the percentage of energy intake from fat is far from recommendation. Evaluation of fat and sodium is given greater weight than others, with a score of 0–3, because these two dietary factors are more related to burden of NCDs in China [22]. Finally, an aggregate score of all the indices will be obtained, which represents the healthiness of whole meal consumption, 0 being the unhealthiest, and 10 being very healthy.

**Table 3:** The key evaluation indices for meal consumption

Evaluation index	Range	Health score	Mark
**Fat**: percentage of energy intake from fat, %	< 20	2	-
	20- < 30	3	Good
	30- < 40	2	+
	40- < 50	1	+ +
	50- < 60	0.5	+ + +
	> 60	0	+ + + +
**Sodium**: actual intake, mg	< 1000	3	Good
	1000- < 1400	2	+
	1400- < 1800	1	+ +
	1800- < 2200	0.5	+ + +
	> 2200	0	+ + + +
**Meat**: actual/recommended intake ratio	< 0.8	1	-
	0.8- < 1.2	2	Good
	1.2- < 1.4	1	+
	> 1.4	0	+ +
**Vegetables**: actual/recommended intake ratio	< 0.5	0	–
	0.5- < 0.7	1	-
	> 0.7	2	Good

### Outcomes

In Phase I, dietary pattern, body weight or blood pressure optimizing is expected after the intervention. The primary outcome for the intervention effectiveness is dietary intakes. Anthropometric indicators including weight, body mass index (BMI), body composition and blood pressure are the secondary outcome. In Phase II, body metabolism normalization is expected after this period.

### Quality control

The quality control team was established prior to this study. All the researchers involved in this study must attend the complete operation training, including study protocol and standard operating procedures of participants’ data collection. In addition, both on-site and on-line review for data verification will be implemented, to ensure the quality and consistency of organizational operations, and stabilize food supply at the canteen.

### Statistical analysis

The Mann–Kendall test will be implemented to examine the temporal trends of lunchtime food supply based on average “traffic light” score and animal/plant food ratio. For dietary record, we hypothesized that at least 5000 person-meal observations would be collected and analyzed over the follow-up period. Generalized linear mixed models with intervention, time and 2-way interaction as fixed factors, will be used to examine the intervention effects on each outcome. A two-sided *p* value < 0.05 will be considered to indicate statistical significance.

### Trial status

The recruitment for the trial was initiated in September 2022, but the field work of Phase I suspended in December 2022 due to the COVID-19 epidemic. Subsequent work is under arrangement and participant recruitment is still ongoing at the time of submission, especially for Phase II. This protocol was completed before the research team had received any data.

## Discussion

To the best of our knowledge, this is a very novel approach providing AI-based dish nutrition evaluation and PN evaluation after meal consumption in order to assist users with nutrition information to make wise food choice in China, which can probably place a dietitian level of nutrition service support, even give timelier tailored diet feedback than dietitians.

In our study, this applet ensures the high quality of evaluation through continuous monitoring of food intakes and physiological indicators, which could demonstrate the potential for scalable use in long-term intervention. Researchers have discovered that PN approach based on Internet or machine learning algorithm could achieve greater reductions in discretionary food intake [23], predict the glycemic responses of individuals to a diverse array of foods [24] and serve as a treatment for obesity and other metabolic diseases [25]. Compared with dietitians, our applet combines multiple data sources, applies big-data analytics and performs AI computing to inform timelier feedback of dish nutrition evaluation and PN evaluation after meal consumption. Notably, the development in smartphone-based telecommunication systems, especially new fifth generation (5G) technology in China with ultrahigh transmission speed, ultralow network latency and enormous data throughput, has improved the prospect of applying this applet to real-time diet management [26].

Detailed recipes with quantity collected by field investigators set a basis for relatively precise calculation of actual meal consumption. The field investigators who receive a standard training course are responsible for weighing raw materials and cooked dishes. In addition, instant dietary record and automatic calculation can be completed through the applet. Compared to traditional dietary survey methods like 3-day 24-h diet record [27], our PN approach would probably avoid recall bias from misreporting individual dietary intake, improving the reliability and validity of study.

We adopted a parallel, randomized, controlled study design in real-life scenario to add credits on translating the trial intervention into real-world practice. Our participants are encouraged to record lunches, follow their daily routines and enjoy real-life meals ad libitum, instead of being forced to eat according to the given diet. During the intervention period, this PN approach recommends a relatively ideal dietary pattern, suggesting the rational intake of food groups, energy and nutrients. This applet has the information related to appropriate nutrition in biological context of the individual, which is the key to driving healthier changes of dietary pattern and physiological status. As a cost-efficient tool for data collection, analysis and visualization, this applet can further expand a variety of inputs including clinical assessments, biomarkers of physiological function and other available data to help researchers optimize the intervention strategy in time and dynamically monitor the effects in the follow-up study [28]. According to results from this population-based trial, more functions for the applet like recommendation for next meal and prediction of health risks, will be developed in the future work based on massive data of historical dietary preference and physiological indicators of individuals.

Admittedly, our study also has several limitations. First, the applet is currently available in Chinese and has not been translated to other spoken languages. Thus it cannot yet be used by non-Chinese speakers. Second, it is almost difficult to blind participants and prevent contamination between intervention and control group since both groups attend the same canteen. Third, food consumed out of the canteen menu are not included in the study analysis since the researchers cannot obtain the full recipes, correspondingly affecting the calculation of complete food intake over the whole day. Finally, real-world situations and meals on free wills would probably bring unpleasant confounders to make the efficacy indistinct. In contrast to the feeding trials with promising results, the impact of the intervention might dilute in real life [29].

Our PN approach has the potential to make up for the lack of dietitian resources to provide nutrition consultation without limitation of time and space, which helps to meet massive nutrition service requirements in China. During the past decade, dining out has become a mainstream lifestyle and catering industry has enjoyed continuous growth in cooking standardization [30, 31]. Our approach, integrating efficacious dietary intervention comprising electronic resources and applying them in a real-world canteen situation, has uniquely fit for Central Food Depot which plays an important role in the standardization of catering chains or related units. If this approach provides indications of public health benefit with reducing dietitians or healthcare professionals’ workload [32], it may make a valuable contribution to other fields of medical research.

## Data Availability

Not applicable.

## Abbreviations

PN: Personalized nutrition
AI: Artificial intelligence
NCDs: Non-communicable diseases
RDs: Registered dietitians
DTRs: Registered dietetic technicians
RCTs: Randomized controlled trials
CNNS: China National Nutrition Survey
PAL: Physical activity level
TEE: Total energy expenditure
REE: Resting energy expenditure
DRIs: Dietary Reference Intakes
BMI: Body mass index
